# Coping and adaptation mechanisms employed by sub-Saharan African migrant women in South Africa

**DOI:** 10.4102/jamba.v11i1.645

**Published:** 2019-06-13

**Authors:** Alice Ncube, Yonas T. Bahta, Andries Jordaan

**Affiliations:** 1 Disaster Risk Management Training and Education Centre for Africa, University of the Free State, Bloemfontein, South Africa; 2 Department of Agricultural Economics, University of the Free State, Bloemfontein, South Africa

**Keywords:** coping and adaptation mechanism, livelihood capitals, migrant women, South–South migration, sub-Saharan Africa, attribute contingent ratings

## Abstract

This article assesses the socio-economic coping and adaptation mechanisms employed by sub-Saharan African migrant women in South Africa using a survey and multi-attribute contingent ratings. The socio-economic and adaptation mechanisms were identified using a sustainable livelihood framework, which included political and cultural capital. This study focused on the rarely investigated South-South migration flows. The results found that the demographic and socio-economic characteristics of migrant women played a significant role in the coping and adaptation mechanisms they employed. Human capital ranked the highest, followed by physical, cultural, social, economic and political capitals. This implies that the livelihood capital has an implication: the migrant women need to have education and health services to survive in day-to-day activities of their life as human capital. They need also to sustain economically at least to cover house rent, food, communicate with family and assist the family as economic and physical capitals. Furthermore, they need to adapt, respect and live with the culture of the host nation in harmony and conducive environment as social, cultural and political capitals.

## Introduction

Globalisation and international migration mark the consistency and pace of contemporary and modern-day society in which people from varied demographic and socio-economic backgrounds migrate everywhere. With globalisation, people including Africans are expanding their exodus destinations because the world is becoming smaller and easily reachable (Rodrigue et al. 2013). International migration to South Africa is not a new phenomenon. Crush (2000) observed that cross-border migration between southern African states and South Africa is nothing new as it dates back to more than 150 years. This influx of migrants was initially to the South African homelands in pre-independence period and after independence (1994) to the main economic centres and hub of the country, such as Johannesburg, Pretoria, Cape Town, Durban and Port Elizabeth, where the main economic activities take place (Fine 2014). Migration is neither gender-blind nor gender-neutral, rather it is gender-sensitive (Ncube 2017). Migrant women are therefore discussed as independent agents of migration; hence, the socio-economic coping and adaptation mechanisms employed by the African migrant women are the central focus.

The region of sub-Saharan Africa (SSA) is depicted as that of people who are very mobile (IOM 2005). Initially, it was mostly unskilled workers who migrated (Taeuber & Eldridge 1947); however, notably since the 1980s, African skilled personnel and professionals, and even African women, joined the flow of migration internationally (Adepoju 2007). A total of 14.5 million international migrants are Africans and of these 10 million moved within the sub-Saharan African region, including South Africa, which is the focus country of this study (Ratha & Shaw 2007). Migration within the African continent is also not easy to document because of the artificiality of the borders which have separated relatives from each other and their movement can be hardly included as international because they can belong to one or more countries. In South Africa, the most migrants are from Zimbabwe (Stats SA 2012). The government report indicated that Zimbabweans in South Africa account for the lion’s share mainly because of Zimbabwe’s proximity to South Africa and for other reasons like ethnic connections, the similarity of culture and the socio-economic and political situation of Zimbabwe. Adams (2003) posited that migration in Africa could be viewed as poverty-limited and poverty-driven as the need for migration depends on the migrants’ resources in their home country.

The movements and configurations of international migration in sub-Saharan Africa like anywhere in the world are triggered by many factors, such as rapid population and excessive human capital, unstable politics, accelerated tribal intolerance, dysfunctional governments caused by unjustified democratisation processes, downward economic development and retrenchment of civil servants because of structural adjustment processes, poverty and, finally but not least, environmental deterioration (Mills & Herbst 2014). According to Bengi-Arslan, Verhulst and Crijnen (2002), challenges related to migration, such as social separation, lack of livelihood and feeling of loss, have been observed as traumatic life events that demand a drastic mindset shift by the migrants in communities. The continuum of vulnerability in migration can impact negatively migrant women in host countries. On the other side, the continuum of resilience can have a positive impact on the same migrant women in the same space (Sudmeier-Rieux 2014). Women have been migrating as single and married (Martin 2004), uneducated and educated entities, dependently and independently in search of livelihoods in other countries for a very long time. Female migration behaviour is not any different from that of migrating men but is influenced by the gendered nature of life. Some would even argue that coping and adaptation is dependent on the gender dynamics of the migration process (Piper 2007).

Existing international and African studies have focused on South–North flows from developing and underdeveloped countries to developed countries (De Haas 2007; Hamilton 1997; Hatton 2004; Khatiwada & Samaniego 2014; Lafleur & Stanek 2017; Rotte, Vogler & Zimmermann 1997; Schiff 1996; Schoorl et al. 2000; Vogler & Rotte 2000). Some studies have focused on South–South migration (Andreopoulos et al. 2011; Facchini, Mayda & Mendola 2013; Gindling 2009; Makina 2012; Melde et al. 2014; Ratha & Shaw 2007). The South–South migration studies have focused on the effect of migration on economic development and security, while the nexus tends to focus on migrant movement, remittances and labour market. None of the South–South migration study has considered migrant women’s coping mechanisms. Therefore, this study attempts to fill this gap in knowledge and literature. Moreover, the novelty of the study lies in the inclusion of political and cultural capitals to the Sustainable Livelihood Framework. The primary objective of the study was to assess the socio-economic and different livelihood capitals in coping and adaptation mechanisms employed by African migrant women in South Africa. The study further analysed the determinant of capitals in their day-to-day survival.

### Conceptual background

The term ‘migrant woman’ is used to refer to all voluntary and involuntary migrant women (Ncube 2017). This includes, *inter alia*, refugees, asylum seekers, students, skilled and unskilled women, businesswomen and wives. The research is grounded on the social network theory, which reinforces the importance of individuals who constitute social networks or groups. The people or groups of people are not passive; they possess social capitals that have been acquired, developed, improved and transferred across generations and societies (Wolff & Moser 2009). The theory emphasises that women are central to accessing networks and mobilising networks, which develop over time within societies. These social networks are built upon values, norms, knowledge, social learning and information sharing that are essential in coping and adaptation dialogue. The social capital is postulated in the Sustainable Livelihood Framework, including physical, financial, political and cultural capitals, as a pillar of the livelihoods that people can utilise in any situation. Therefore, the social theory was used as the conceptual basis for the study.

The Sustainable Livelihood Framework portrays how the vulnerability context outlines people’s ability to survive and earn a living, which may result in them engaging in migration. However, the livelihood capitals such as the human, natural, social, financial and physical capitals can also influence the vulnerability context. The people may lack certain capitals and, together with how they are exposed to hazards, this can determine if they will be able to survive in the current situation or may be forced to migrate. In this same light, the policies, processes in the form of government and other structures in place, laws, regulations and the culture of the specific community also have an influence on the survival and overall well-being of the people. These policies and institutional processes also affect the coping and adaptation strategies devised, and therefore have an influence on the ultimate livelihood outcomes of a community (Hugo 2005). The Sustainable Livelihood Framework basically puts emphasis on the strategy for poverty reduction, survival and prosperity that is dependent on the ability of individuals or a community to capitalise on the opportunities and resources at their disposal. These livelihood activities may be in the form of socio-economic goods and services (Adato & Meinzen 2002). Scoones (1998) and Majale (2002) stated that the Sustainable Livelihood Framework is versatile as it can be applied at individual, household, community and region or even at the country level.

Coping according to Folkman and Moskowitz (2004) represents the beliefs and actions used to accomplish internal and external demands of situations, which are stressful. Migrants experience some form of stress in the environment of the new country (Bhugra & Becker 2005). Adaptation is a process of modification and adjustment to new environmental conditions (Berry et al. 2002). Coping and adaptation of migrant women is, therefore, the process of fitting into the society of a community and functioning successfully in a host environment. Migrant women, like any other migrants, encounter a ‘new-born’ condition and difficulties in a new host environment. They are in the new environment as individuals or with other people close to them, but still they have to experience the new environment as individuals. Some of these difficulties could be more traumatic for migrant women, especially if they come to the host country as dependents or independents, with ‘less’ education, being unprepared, encounter discrimination and lack in the necessary language skills (Çakir 2009). It is worse if these migrant women are trafficked into the host country against their will as they may be beginning another life of modern-day slavery, which has been witnessed in various parts of the world.

According to Çakir (2009), Turkish women migrants in the United Kingdom (UK) are doubly disadvantaged by the mere fact that they are female, and more so when issues of ethnicity, race, class or religion are brought to the fore. However, such vulnerabilities differ from one woman to another depending on certain factors, their resilience mechanisms and physiognomies they possess. Migrant women may utilise the demographic characteristics that they have. Conversely, they may find difficulty in negotiating general well-being (Gsir 2014). They might encounter relationship losses, lower life status and isolation, which make life unbearable for them (Bhugra & Becker 2005). Migrant women may also be victims of precarious work which is characterised by high flexibility and poor levels of, or none, social protection (Pajnik 2016). Migrant women’s levels of resilience vary and are influenced by their demographic and socio-economic characteristics, some of which are age, educational level, primary position in the household, occupation, marital status and entry permit.

More than 80% (268) of the respondents were in the age group of 18–49 years. The respondents fell in the productive age group and this facilitated their coping and adaptation abilities (Van der Ven & Smits 2011). This implied that the respondents were able to earn a livelihood and to fend for themselves. They could work in any job, that is, they might be adjusted to any condition such as selling in the streets or even working at salons and people’s house. Their educational levels meant that they could negotiate their livelihoods better than non-educated ones. The adversity factors because of the gendered nature of migration can also be taken as a disempowering experience, which can result in a particular physical or social vulnerability for an individual migrant woman. For instance, factors like educational level (Berry, Sam & Berry 2006) and language aptitude (Beiser & Hou 2001) can be taken as important contributing factors for women migrants’ positive adjustment. International migration in sub-Saharan Africa is resonant and at the same time not easy to understand as most of the migrants moving into South Africa consider the country to have the most developed economy in Africa (Adepoju 2006). This was echoed by Campbell (2013) who highlighted that South Africa is a preferred and a perceived destination for many African migrants because of the perception that the country has a thriving and vibrant economy.

## Material and methods

South Africa is a preferred area to study migration by the researchers because compared to other African countries, South Africa is perceived by many African fellows as the most developed country and easy to open a small business and improve livelihood. The exact number of migrants in South Africa is unknown as there are many illegal and undocumented migrants in the country (Tati 2008). However, in 2015, Statistics South Africa estimated illegal migrants in the country to be between 500 000 and 1 000 000. Female migrants are also a common feature in many regions of the world, and particularly in South Africa (Hiralal 2017). In this study, 332 African migrant women were surveyed over 3 months (February 2016 – April 2016) and they included women from six metropolitan cities in South Africa.

**FIGURE 1:**
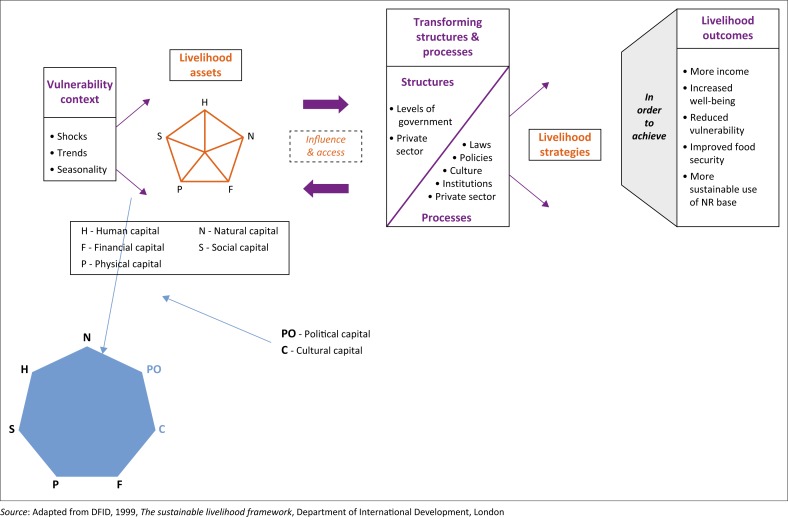
Sustainable Livelihood Framework.

In this study, a multiple-stage sampling technique was employed. The first stage entailed the selection of the four out of the nine provinces of South Africa. The provinces selected were the Free State, Gauteng, KwaZulu-Natal and Western Cape. The three later provinces were selected on the basis that they were the economic hubs of the country. Conversely, the Free State province was selected because of its accessibility and availability of respondents to the researcher. The next stage of sampling was the ballot choice of the metropolitan cities. The six metropolitan cities that were randomly selected were Bloemfontein in the Free State province, Johannesburg, Pretoria and Ekurhuleni all from Gauteng province, Durban from KwaZulu-Natal province and Cape Town from the Western Cape Province. A questionnaire was used to collect data on respondents’ demographic, socio-economic characteristics and socio-economic coping and adaptation mechanisms. In this study, a Sustainable Livelihood Framework (see Figure 1) was used to identify variables and interpret results by the inclusion of political and cultural capitals.

The socio-economic coping and adaptation mechanisms used by migrant women were categorised into four themes: family support, entrepreneurial support, employment and humanitarian support. To determine the most prominent socio-economic coping and adaptation mechanisms for African women migrants, multi-attribute contingent ratings of the identified survival mechanisms were conducted using mean scores estimated from the sampled respondents. Similarly, the multi-attribute contingent ratings using mean scores were used to determine factors that influence the choice of socio-economic coping mechanism and the adaptation thereof by African women migrants. The multi-attribute contingent ratings approach was used to rate the human, social, economic, physical, political and cultural livelihood capitals to ascertain the most evident indicators that influenced African migrant women’s coping and adaptation in South Africa.

Making use of the multi-attribute contingent ratings of the livelihood capitals and capital factors, the mean scores of the respondents gave the indication as to what the migrant women utilised in order to cope and adapt in South Africa. The livelihood capitals and the factors included the following:

- Human: education, health, knowledge and skills and capacity to work
- Social: networks and connections, relations of trust and mutual support, and informal and formal networks
- Economic: salary, wages, income, savings, credit, pension, marriage, *stokvels*,^1^ and burial societies
- Physical: shelter and/or houses, amenities (basic services); communication and technology: Internet, emails, cell phones, vehicles, transport networks, etc.
- Political: relationships with political authorities in South Africa, political networks from home country, interaction with political structures locally and business relations with politicians
- Cultural: gender issues, traditional beliefs, work ethics and respect for authority.

### Ethical considerations

Ethical clearance was sought from the University of the Free State (tracking number: UFS-HSD2016/0037).

## Results and discussion

### Socio-economic characteristics of the respondents and coping and adaptation mechanisms

The demographic and socio-economic characteristics of migrant women were the main determinants of coping and adaptation in host communities (Warfa et al. 2012). The demographic and socio-economic characteristics of the respondents are presented in Table 1. The respondents in the study were from Angola (1), Benin (1), Burundi (5), Cameroon (16), Congo-Brazzaville (1), Democratic Republic of Congo (DRC) (16), Eritrea (1), Ethiopia (3), Ghana (12), Ivory Coast (1), Kenya (3), Lesotho (7), Malawi (6), Mauritius (1), Mozambique (10), Nigeria (29), Rwanda (2), Senegal (1), Somalia (1), Tanzania (1), Uganda (4), Zambia (4) and Zimbabwe (187). All the respondents were above 18 years of age (one respondent did not disclose her age). The migrant women entered South Africa under various visas or permits, mainly visitors’ visas, study permits and some entered the country illegally. A third (33.43%) of respondents were aged between 18 and 29 years. Twelve (3.61%) respondents were aged between 50 and 59 years and only three (0.9%) respondents were aged 60 years or above (two from Zimbabwe and one from Somalia). Forty-eight respondents (14.46%) were aged between 40 and 49 years. One hundred and fifty-seven (47.29%) respondents were aged between 30 and 39 years, which is considered to be a highly productive age group (Bongaarts 2001). This could contribute to their coping and adaptation mechanisms in South Africa. The high ratio of the productive or working-age people compared to the total sample clearly indicates their impact on economic growth, which could make women capable of looking after themselves and contribute to the economy directly or indirectly (Van der Ven & Smits 2011). This implied that the respondents were able to earn a livelihood and manage to cope and adapt, without burden to the government, some of them even being able to create a job for local nationals.

**TABLE 1:** Respondents’ socio-economic and demographic characteristics.

Characteristic	Total respondents	%
**Age (year)**		
18–29	111	33.43
30–39	157	47.29
40–49	48	14.46
50–59	12	3.61
60+	3	0.90
Others (those who do not disclose)	1	0.31
**Marital status**		
Single	105	31.63
Staying with partner	2	0.60
Engaged	1	0.30
Married	173	51.11
Widowed	21	6.33
Divorced	9	2.71
Separated	8	2.41
Never married	11	3.31
Others (those who do not disclose)	2	0.60
**Education**		
Lower primary (Grade 3/Standard 1)	2	0.60
Upper primary (grades 4–7/Standards 2–6)	21	6.33
Secondary	193	58.13
Certificate	25	7.53
Diploma	36	10.84
Technikon qualification	5	1.51
University degree	18	5.42
Postgraduate qualification	31	9.34
No education	1	0.30
**Proficiency in English**		
Yes	311	93.67
No	21	6.33

The marital status of the respondents impacted both negatively and positively how they coped and adapted in South Africa. More than half of the respondents (52.11%) were married, 31.63% were single and the remaining were either cohabiting (0.6%), engaged (0.3%), widowed (6.33%), divorced (2.71%), separated (2.41%) or never married (too old to be considered as a single) (3.31%). As about 52.11% of the respondents were married, this could, therefore, have been used as a coping and adaptation mechanism. Sánchez-Domínguez, De Valk and Reher (2011) found that endogamy was considered by migrants from Morocco, but not from Ecuador, Argentina, Romania and Colombia, as a viable coping strategy soon after arriving in Spain. Educated migrants who have the university degree and postgraduate qualifications (15% of the respondents) in this study did not prefer endogamous marriages, but they had nothing against marriage. This was correlated with the way the respondents valued marriage as a coping mechanism.

One hundred and two (30.7%) respondents indicated that they valued marriage as a coping mechanism to a small extent, while 86 (25.9%) indicated that they did not value marriage at all as a survival mechanism because they did not benefit from marriage. Gsir (2014) confirmed that marriage could be useful for coping and adaptation because Pakistan migrant women who migrated as spouses to Britain utilised marriage to ensure safe passage. Evidence of utilisation of the marriage to cope and adapt was further portrayed by the Asian women who migrated to rural South Korea to marry and in doing so addressed issues of declining birth rate and the ageing population (Kim, Kim & Joh 2015). Those migrant women were assured of better welfare, safety and better coping and adaptation by marrying a wealthy husband in order to fulfil their needs.

Levels of education were important for the coping and adaptation mechanisms employed by the respondents. Education was an important human capital used as a coping mechanism. More than half of the respondents (58.13%; 193 out of 332) had a secondary education, 7.53% had certificates, 10.84% had diplomas, 1.51% had a technikon qualification, 5.42% had university degrees and 9.34% of respondents had university degrees and postgraduate qualifications. One respondent from the DRC had no education at all. She indicated that her five children who migrated with her from DRC after fleeing political turmoil looked after her.

It is imperative for migrant women or any migrants to have certain levels of linguistic capital in the language(s) of the host country in order for to be able to communicate (Madziva, McGrath & Thondhlana 2016). English is one of the main languages used in South Africa. However, there are ten more official languages in South Africa. Knowledge of the official language is an instrument for integration and acculturation (Celenk & Van de Vijver 2011). This could assist migrant women in getting employment and acquiring relevant papers in order to get employment or to even start businesses. Most of the respondents (76.8%) stated that the official language in their home countries was English, followed by French (62) and Portuguese (11). Amharic was used by three Ethiopians and Tigrigna by an Eritrean. Those languages had links of the countries of origin whereas English, French and Portuguese had their origins in the historical links to colonial countries of Britain, France and Portugal (Stuchtey 2011).

On arrival, the respondents had to cope and adapt by using English, which is the main business language in South Africa, or they had to learn of the other ten official languages. Three hundred and eleven (93.67%) respondents indicated that they were proficient in English language and only 21 (6.33%) were not. The proficiency level in the English language suggested that the ability to communicate in English was a coping and mechanism. In South Africa, English is the dominant language of the economy and government (Casale & Posel 2011). Being proficient in the dominant language reduces transaction and information costs of an individual trying to negotiate the terms of employment and access the job market (Casale & Posel 2011). Besides being proficient in English, the respondents were able to speak and understand a number of South African local languages, namely, Afrikaans, Zulu, Xhosa, Southern Sotho, Northern Sotho, Pedi, Ndebele, Venda, Tswana and Swati. These languages made the lives of the migrant women easier as they were able to integrate into the communities. As indicated in Table 2, 130 respondents stated that they spoke Zulu and 33 migrant women stated that they spoke Tswana. Zulu was the most understood local language, with 183 respondents indicating that they understood the language. A total of 126 respondents indicated that they understood Xhosa, 58 understood Northern Sotho, 78 understood Southern Sotho, 28 understood Afrikaans, 51 understood Tswana, 54 understood Ndebele, 24 understood Tsonga, 30 understood Venda and 15 understood Swati. Of the 78 respondents in Durban, 45 spoke Zulu and 32 spoke Xhosa; in Bloemfontein, 48 of the 82 respondents indicated that they spoke Southern Sotho; and in Johannesburg, Pretoria and Ekurhuleni metropolitan cities, respondents spoke a number of local languages. For instance, 12 of the 15 respondents spoke Zulu in Ekurhuleni. As stated by Krumm and Plutzar (2008), migrants become aware that for them to fit in and be accepted in host communities they needed to understand local languages fully.

**TABLE 2:** Respondents who understood local languages.

Language	Understand				Did not say	Total
	Yes		No			
	Number of respondents	Valid percentage (%)	Number of respondents	Valid percentage (%)
Zulu	183	55.79	145	44.21	4	332
Xhosa	126	38.41	202	61.59	4	332
Northern Sotho	58	17.68	270	82.32	4	332
Southern Sotho	78	23.78	250	76.22	4	332
Afrikaans	28	8.56	299	91.44	5	332
Tswana	51	15.55	277	84.45	4	332
Ndebele	54	16.62	271	83.38	7	332
Tsonga	24	7.32	304	92.68	4	332
Venda	30	9.20	296	90.80	6	332
Swati	15	4.59	312	95.41	5	332

Being able to communicate in the host country languages can be the main driver of social and economic integration and assimilation for migrant women in South Africa. The study found that Ghanaian, Nigerian, Zimbabwean and Congolese respondents had businesses especially in the hairdressing industry. Their main clients and employees were locals, especially black South Africans. Understanding local languages constituted a vital part of the respondents’ human capital. Other personal spheres were affected by knowledge of local languages and included health, education, marriage, social integration and political participation. Higher levels of local language proficiency correlated with the respondents’ socio-economic coping and adaptation in South Africa.

### Livelihood capital factors as survival mechanisms

#### Human livelihood capital factors as survival mechanisms

Four human capital factors were identified: education, health, knowledge and skills, and capacity to work. The respondents were asked to assign a score for each of the factors that made them cope and adapt in South Africa. Over half (52.4%) of the respondents ranked education as very high, 16.6% ranked education as high and 16.6% ranked education as moderate. Thus, 276 (83.1%) of the respondents indicated that education was a coping mechanism. Those who ranked education as very low (9%) stated that they had jobs, which did not require an education.

There was one respondent selling vegetables in Parow, Cape Town, who stated that she was a qualified school teacher but because she could not be employed by the Department of Education, she resorted to selling vegetables. Thus, some respondents deskilled themselves in order to survive in South Africa. This was also the case in Canada where migrant women had to accept lower jobs than what they were qualified for in order to earn a livelihood (Galabuzi 2006).

A total of 83.1% respondents indicated that health was very central to their coping and adaptation in South Africa. They indicated that they were physically and mentally healthy and those of them who had once fallen sick were treated at various public and private health institutions in South Africa. Generally, they praised the public health system of the host country. This was because of the proportion of the population that is covered by the South African public health care system (World Health Organization [WHO] 2007). The respondents confirmed that the health services are accessible and affordable as compared to their countries of origin. However, in Johannesburg, Pretoria and Ekurhuleni, the respondents voiced dissatisfaction with the manner and treatment they received from public health facilities. Respondents mainly from Zimbabwe expressed their concerns as they were publicly ridiculed for causing overcrowding in their hospitals. One respondent stated that one health worker said, ‘*Buyela kini, niyasiminya*’ (‘Go back to your country, you are suffocating us’).

Most of the respondents (76.8%) considered knowledge and skills to be very important for survival in South Africa. Respondents who entered on study permits managed to obtain an education and others managed to upgrade and get higher qualifications, thereby increasing their human capital value. That would have enabled them to increase their bargaining power in the work market and to improve their remuneration and working conditions (Dustmann 2003). Respondents were either entrepreneurs, employed or supported by family and friends. They utilised their skills and knowledge to earn a living. The respondents further explained that they used their skills to engage in self-employment, menial jobs like housekeeping and sales assistants. Those who worked in salons used their talents and skills they learnt in their home countries to earn money. Some women expressed joy in the fact that the housekeeping jobs they were doing in South Africa was not too difficult as they made use of machinery like carpet cleaners, washing machines and food processors unlike in their home countries.

Table 3 outlines how respondents rated themselves in terms of human capital factors. The majority of the respondents ranked human capital factors as very high. The ratings were education (52.7%), health (83.4%), knowledge and skills (76.8%) and capacity to work (82.8%). This ranking was in line with the levels of education, their health status, their capacity to work and knowledge and skill that the respondents possessed.

**TABLE 3:** Ratings of the human capital factors (*n* = 332).

Human capital factor	Very low		Low		Moderate		High		Very high		Mean score	Factor ranking
	*n*	%	*n*	%	*n*	%	*n*	%	*n*	%		
Education	30	9.0	26	7.8	55	16.6	46	13.9	175	52.7	3.93	4
Health	2	0.6	6	1.8	19	5.7	28	8.4	277	83.4	4.72	1
Knowledge and skills	4	1.2	11	3.3	24	7.2	38	11.4	255	76.8	4.59	3
Capacity to work	4	1.2	9	2.7	13	3.9	31	9.3	275	82.8	4.70	2

Health was ranked the highest human capital factor because of the use of the perceptions as well as the experiences of the respondents that the South African health system was one of the best in the continent. Capacity to work was ranked second, followed by knowledge and skills and finally education.

#### Social capital as a survival mechanism

Social factors that assisted respondents’ survival were networks and connections, relations of trust and mutual support, informal and formal networks and, finally, collective representation. Formal and informal groups were identified as very low in terms of coping and adaptation in South Africa. Table 4 indicates the rating of the social livelihood capital factor.

**TABLE 4:** Ratings of the social livelihood capital factors (*n* = 332).

Social livelihood capital factor	Very low		Low		Moderate		High		Very high		Mean score	Factor ranking
	*n*	%	*n*	%	*n*	%	*n*	%	*n*	%		
Networks and connections	59	17.8	27	8.1	47	14.2	26	7.8	173	52.1	3.68	2
Relations of trust and mutual support	41	12.3	35	10.5	64	19.3	35	10.5	157	47.3	3.70	1
Formal and informal groups	113	34.0	37	11.1	52	15.7	31	9.3	99	29.8	2.90	4
Collective representation	87	26.2	24	7.2	81	24.4	31	9.3	109	32.8	3.15	3

A total of 52.1% of respondents indicated that they considered networks very high as a survival mechanism. Relations of trust and mutual support that the respondents gained from their relations in South Africa also assisted them to cope and adapt. A total of 192 (57.8%) respondents rated the relations based on trust and mutual support both high and very high. Networks mentioned by respondents included church, family, friends, ethnic groups, political connections, colleagues and neighbours. Fifty-nine (17.8%) of respondents believed that networks and connections were necessary for their lives and had been instrumental in their survival in South Africa. For instance, family support and spousal support assisted respondents to cope on arrival in South Africa and those relationships also made the women acculturate better in the new environment. This was in agreement with the findings by Ibañez et al. (2015) who stated that local inhabitants developed relations with foreign women when they were in their employment. A number of respondents from Ghana (10), DRC (6), Mozambique (4) and Zimbabwe (25) who were salon owners stated that they employed locals as well and they worked well together. Good rapport was developed among the respondents and locals, which made coping and adaptation easier. There were respondents in Durban (4 from Zimbabwe, 3 from DRC and 2 from Nigeria), however, who expressed that they did not trust anyone and also felt alienated in South Africa. Those same respondents even expressed that they would be glad if they got means of returning back to their home countries. Those women had bad experiences of xenophobic attacks, had been denied employment because of being a foreigner or had ‘… their husbands snatched by local women’.

In the workplace, formal groups were established, and in the communities informal groups were formed. However, 34% of respondents had a very low rating for formal and informal groups as coping mechanisms in South Africa. That related well to trust and mutual support issues where many respondents expressed verbally that it was difficult to rely on others to earn a livelihood. On the other hand, 29.8% of respondents rated those formal and informal groups as very high.

Approximately, one-third of respondents rated collective representation as very high (32.8%), with 9.3% rating high. The respondents indicated that they used their home country connections, like the Zimbabwe Association in South Africa and Cameroonians in South Africa, to represent them when they were not fairly treated in South Africa. Most of the respondents (85%) indicated that they utilised religious organisations such as the church as a network means to survive, to help each other in bad time and their view of the church as a good surviving mechanism ranges from a small to large extent. Collective representation as a social capital was useful in coping and adaptation in their host country.

#### Economic capital as a survival mechanism

Six economic factors were identified: salary/wages/ income, savings, credit, pension, marriage and, finally, *stokvels* and burial societies. Overall, the respondents did not indicate economic capital as a strong capital that they employed to cope with and adapt in South Africa. The reason for that was because they had to utilise any available means to survive regardless of their other capitals, especially the human capital. The respondents had to deskill themselves in order to survive. Some had to accept low-paying jobs. They did not have much bargaining power in the work sphere because they lacked the requisite permits and because they were women. One woman from Nigeria indicated that she was denied an accounting position because she was a foreigner and also that she was a woman. The women who were employed therefore rated this factor as moderate, which indicated that they were not really happy about their financial situation although they were surviving in South Africa.

Of the respondents, 38% ranked savings as high and 16.9% ranked as very high (see Table 5). This indicated that there were not many (38.3% or 127 respondents) migrant women who had savings in South Africa. Most of the women (127 and 52) rated savings as very low and low, respectively. When asked to explain their ratings, they stated that their earnings were for daily survival and not for saving. There was also a strong indication that the women were remitting some of their earnings to their home countries. Credit facilities were available in South Africa and those facilities were useful for the economic well-being of people. However, 78.6% of respondents rated access to credit as very low for them because they did not have guarantees, or even insurances as collateral security to access credit. They had no access to the credit facilities offered by financial institutions in the country because of a variety of reasons. Some of the reasons included incorrect documents and no collateral evidence needed by the banks. Two hundred sixty-one (78.6%) of the respondents indicated that they were not used to credit as they were accustomed to paying cash for goods and services.

**TABLE 5:** Ratings of the economic livelihood capital factors (*n* = 332).

Economic livelihood capital factor	Very low		Low		Moderate		High		Very high		Mean score	Factor ranking
	*n*	%	*n*	%	*n*	%	*n*	%	*n*	%		
Salary/wages/income	39	11.7	80	24.1	122	36.7	38	11.4	53	16.0	2.96	2
Savings	127	38.3	52	15.7	59	17.8	38	11.4	56	16.9	2.53	3
Credit	261	78.6	21	6.3	25	7.5	14	4.2	11	3.3	1.47	5
Pension	297	89.5	9	2.7	4	1.2	9	2.7	13	3.9	1.29	6
Marriage	135	40.7	15	4.5	26	7.8	19	5.7	137	41.3	3.02	1
Stokvels/burial societies	203	61.1	23	6.9	17	5.1	23	6.9	66	19.9	2.17	4

From Table 5 it is clear that salaries were rated by the respondents as low (24.1%) to moderate (36.7%). More than one-third of the respondents (38.3%) indicated that their savings were very low and only 16.9% rated their savings as very high. Economists encourage individual level and household savings that benefit individuals and their families. Under-saving or not saving at all may influence variable consumption and low resilience to shocks (Karlan, Ratan & Zinman 2014). Migrant women could be vulnerable because they are not saving, but as groups, the migrant women are investing in their home countries and also using the money to educate their families and themselves (Dayton-Johnson et al. 2009). The rating of the *stokvels* and burial societies was very low at 61.1% because most burial societies cater for local burials. Most foreigners repatriate their compatriots’ remains and they rely on their home country associations to do that.

Respondents did not believe in pensions, with 89.5% rating pensions as very low. They considered pensions to be long-term wishes, and instead focused on immediate wishes like educating their children and remitting money home to invest there. They were also some who were saving money so that they could return to their home countries and open up businesses. For example, respondents from DRC expressed their wish to return to their country and open businesses there one day when true democracy was attained. This would be highly unlikely in the foreseeable future as the socio-economic and political conditions in DRC worsened as was reported in the 2014 Human Development report that the country was the second from last in global living conditions (UNDP 2014).

Marriage as an economic factor showed an interesting trend, with 40.7% respondents ranking very low and 41.3% ranking very high. The respondents gave various reasons for ranking marriage very low. Reasons included ill-treatment by husbands in South Africa, and husbands or partners not willing to take up any kind of job in order to contribute towards the household income. Those who ranked marriage very high were happy in their marriages and some respondents based their ranking on biblical values that the marriage institution is held high in providing for the family. The respondents mostly ranked *stokvels* and burial societies as very low (61.1 %), with 19.9 % rating them as very high. They did not use burial societies and *stokvels* because the societies catered for local burials and the respondents wanted to bury their loved ones in their country of origin. The respondents had their own kinship associations that assisted them when they were bereaved and they expressed their appreciation of compatriots who helped during bereavement.

**FIGURE 2:**
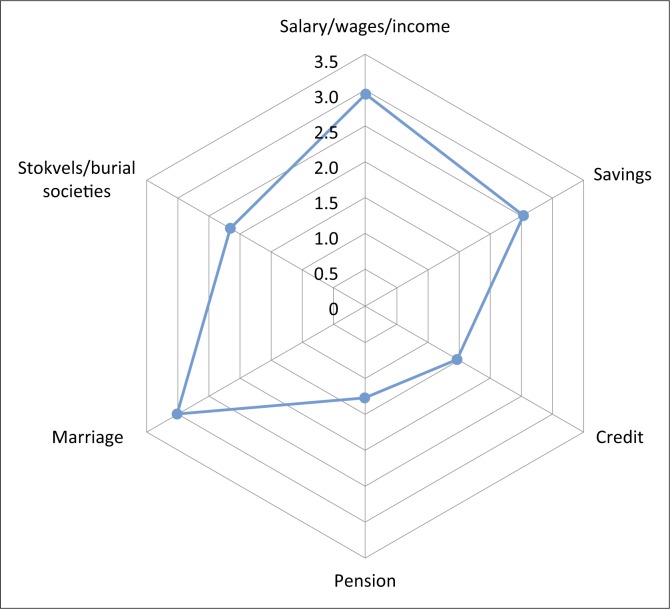
Economic factors of survival.

Figure 2 indicates marriage as an economic factor for survival and it was ranked the highest. One hundred and eighty-one respondents perceived those married to be better placed in the economic sphere because a number of them had not managed to gain employment compared to their male counterparts. Two hundred and thirty-one respondents indicated that the marriage institution was important to them regardless of their marital status because they believed those who were married received social protection from their spouses together with the economic benefits. The respondents expressed that various kinds of marriages such as customary law marriages, civil marriages or unions, religious marriages and common law marriages were recognised in South Africa. This indicated how the well-being of the people, especially women, were taken care of by laws that protect them contrary to what they experienced in their home countries where some have been deprived of inheritances because of the marriages they were in.

#### Physical capital as survival mechanism

The four factors of the physical capital identified in this study included shelter/houses, amenities (basic services), communication services (vehicles, transport networks) and technology (Internet, emails, cell phones, etc.). The respondents were all very happy about the physical capital in South Africa. This included the houses they lived in, basic services like water, sanitation and hygiene provisions, commuting services and technological systems. Table 6 shows that the majority of the respondents rated all the four physical factors as very high.

**TABLE 6:** Ratings for physical livelihood capital factors (*n* = 332).

Physical livelihood capital factor	Very low		Low		Moderate		High		Very high		Mean score	Factor ranking
	*n*	%	*n*	%	*n*	%	*n*	%	*n*	%		
Shelter/houses	15	4.5	39	11.7	36	10.8	51	15.4	191	57.5	4.10	4
Amenities (basic services)	10	3.0	23	6.9	35	10.5	43	13.0	221	66.6	4.33	2
Communication services	11	3.3	17	5.1	38	11.7	41	12.3	224	67.5	4.36	1
Technology	17	5.1	21	6.3	34	10.2	29	8.7	231	69.6	4.31	3

All the respondents (332) were residing in formal houses and none stayed in the informal settlements even though they were renting the accommodation from local South Africans. The respondents who were staying in townships mainly occupied Rural Development Programme houses. These houses were, however, constructed to enable South African citizens to live in decent houses (Pillay et al. 2008). The respondents indicated that they were either renting or bought the houses from the South African citizens.

#### Political capital as a survival mechanism

The political capital issues that were faced by the respondents were relationships with political authorities in South Africa, political networks from home country, interaction with local political structures and business relations with politicians. The respondents indicated that their relationship with the political structures in South Africa was very low, with 91.3% ranking their relations as very poor. As indicated in Table 7, political capital did not contribute much to the coping and adaptation mechanisms of the respondents. The respondents expressed their dissatisfaction with the political situation both in their home countries and in South Africa. They even blamed local politicians for instigating violence against them. An example of that is the 2015 xenophobic attacks, which many people believed were initiated by the King Goodwill Zwelithini in KwaZulu-Natal. Hamber and Lewis (1997) said that violence was seen to be an appropriate way of achieving goals, especially as the violence was legitimised by most political role players. According to Harris (2001), the apartheid government in South Africa legitimised violence as a way of dealing with the then oppressed black local South Africans. This was later legitimised by the new dispensation after independence (Harris 2001). Violence in South Africa is rife, especially service delivery-related violence. Lack of basic service provision by municipalities results in communities embarking on violent protests which were rife during apartheid times. These protests are later turned into xenophobic attacks as foreigners are soft targets and are also blamed for the lack of services by local. This made coping and adaptation difficult for the migrant women as safety institutions in the form of government leaders including traditional leaders were not offering much support.

**TABLE 7:** Ratings of political livelihood factors (*n* = 332).

Political livelihood capital factor	Very low		Low		Moderate		High		Very high		Mean score	Factor ranking
	*n*	%	*n*	%	*n*	%	*n*	%	*n*	%		
Relationship with political authority in South Africa	303	91.3	11	3.3	10	3.0	5	1.5	3	9.0	1.17	3
Political network from home country	289	87.0	15	4.5	13	3.9	6	1.8	9	2.7	1.29	1
Interaction with local political structures	303	91.3	11	3.3	10	3.0	2	0.6	6	1.8	1.18	2
Business relations with politicians	308	92.8	14	4.2	5	1.5	4	1.2	1	0.3	1.12	4

Politics seems to be the biggest let-down for many Africans including the migrant women in South Africa. Example includes the current unsettle political issues in Zimbabwe, DRC, Cameroon, Somalia, Eritrea and Ethiopia. However, the current development in politics in Eritrea and Ethiopia seems a positive development towards peace and prosperity. Many migrants left their home countries because of political challenges and poor governance issues. Poor governance in many sub-Saharan countries contributes to the unprecedented migration of people to South Africa (Alonso 2011). Poverty, because of bad governance by politicians and ill-management of natural resources (Surkin 2011), has resulted in people’s livelihoods being depleted. For instance, migrants from DRC and Somalia leave their country because of bad governance and purposely created conflicts with neighbour countries in order to stay in power for long.

#### Cultural capital as a survival mechanism

Cultural capital factors included gender issues, traditional beliefs, work ethics and respect for authority (see Table 8). Most of the respondents ranked cultural livelihood capitals as very high, with the exception of traditional beliefs (35.2% giving a very low ranking).

**TABLE 8:** Ratings of the cultural livelihood capital factors (*n* = 332).

Cultural livelihood capital factor	Very low		Low		Moderate		High		Very high	
	*n*	%	*n*	%	*n*	%	*n*	%	*n*	%
Gender issues	37	11.1	29	8.7	50	15.1	27	8.1	189	56.9
Traditional beliefs	117	35.2	31	9.3	28	8.4	19	5.7	137	41.3
Work ethics	30	9.0	31	9.3	69	20.8	40	12.0	162	48.8
Respect for authority	35	10.5	17	5.1	28	8.4	28	8.4	224	67.5

Even if the respondents’ responses regarding the gender issue as a category were moderate, low, high, very high and very low, almost all of the respondents were happy with the way gender issues were handled in South Africa. They expressed that authorities paid attention to gender equality. They expressed that there was more respect for women in South Africa compared to their countries of origin, especially by the South African policymakers. Hence, 56.9% of respondents rated gender issues as very high and 27.7% rated as high. When asked why they rated gender issues as such, they expressed satisfaction with the way the cases of gender-based violence were handled by the relevant institutions and the communities they lived in. The democracy as protected in the Constitution of South Africa (1996) (South Africa 1996) allowed the migrant women to enjoy the cultural capital to the fullest. According to the Constitution, Section 9 allows for equality, Section 15 is concerned for the freedom of religion, belief and opinion, and Section 18 deals with the freedom of association. These three pieces of legislation make it easier for migrant women to worship and be associated with any religion, traditional beliefs of their choice.

### Overall ranking of the livelihood capital factors as coping and adaptation mechanisms

Table 9 depicts the overall socio-economic coping and adaptation mechanisms that were identified and employed by the respondents. Six livelihood capitals were evaluated and ranked according to what the respondents possessed. The results indicated that human capital ranked the highest, followed by physical, cultural, social, economic and political capitals. Human capital can be converted into economic capital as migrants contribute to the country’s gross domestic product through various taxes. A healthy and knowledgeable human capital could be beneficial to South Africa. Self-employed migrant women not only come to South Africa with a high level of skills and knowledge, but also provide the much-needed ‘capital brings capital investment’ (Dayton-Johnson et al. 2009). Hence, the ratings of the capital factors ranged from (1) health, (2) capacity to work, (3) knowledge and skills and (4) education.

**TABLE 9:** Capital factors helping women migrants to cope and adapt in South Africa.

Number	Indicator/factor	Rating score	Capital	Mean score
1.	Education	3.93^4th^	Human	4.49^1st^
	Health	4.72^1st^		
	Knowledge and skills	4.59^3rd^		
	Capacity to work	4.70^2nd^		
2.	Networks and connections	3.68^2nd^	Social	3.36^4th^
	Relations of trust and mutual support	3.70^1st^		
	Formal and informal groups	2.90^4th^		
	Collective representation	3.15^3rd^		
3.	Salary/wages/income	2.96^2nd^	Economic	2.24^5th^
	Savings	2.53^3rd^		
	Credit	1.47^5th^		
	Pension	1.29^6th^		
	Marriage	3.02^1st^		
	Stokvels/burial societies	2.17^4th^		
4.	Shelter/houses	4.10^4th^	Physical	4.28^2nd^
	Amenities (basic services)	4.33^2nd^		
	Communication services	4.36^1st^		
	Technology	4.31^3rd^		
5.	Relationship with political authority in South Africa	1.17^3rd^	Political	1.19^6th^
	Political network from home country	1.29^1st^		
	Interaction with political structures locally	1.18^2nd^		
	Business relations with politicians	1.12^4th^		
6.	Gender issues	3.91^2nd^	Cultural	3.75^3rd^
	Traditional beliefs	3.08^4th^		
	Work ethics	3.82^3rd^		
	Respect for authority	4.17^1st^		

Note: Rating score superscript 1st, 2nd, 3rd, 4th, 5th and 6th refers to ranking of capital factors.

In terms of physical capital factors, the respondents were extremely happy with the various communication systems at their disposal such as public and private transport infrastructure, connectivity networks like cell phones, Internet and other social communication networks. The respondents also acknowledged the accessibility of shelter. Hence, the ratings of the physical capital factors were as follows: (1) communication services, (2) amenities/basic services, (3) technology and (4) shelter/houses.

With political capital factors, the respondents expressed their near dissociation with the political structures from both their home countries and South Africa. This was because of the fact that political challenges were the root cause of most of the respondents’ migration to South Africa. In South Africa, they also faced challenges associated with political structures. Hence, the ratings of the multiple political capital factors were as follows: (1) political from home country, (2) interaction with political structures locally, (3) relationship with political authority in South Africa and (4) business relations with politicians.

**FIGURE 3:**
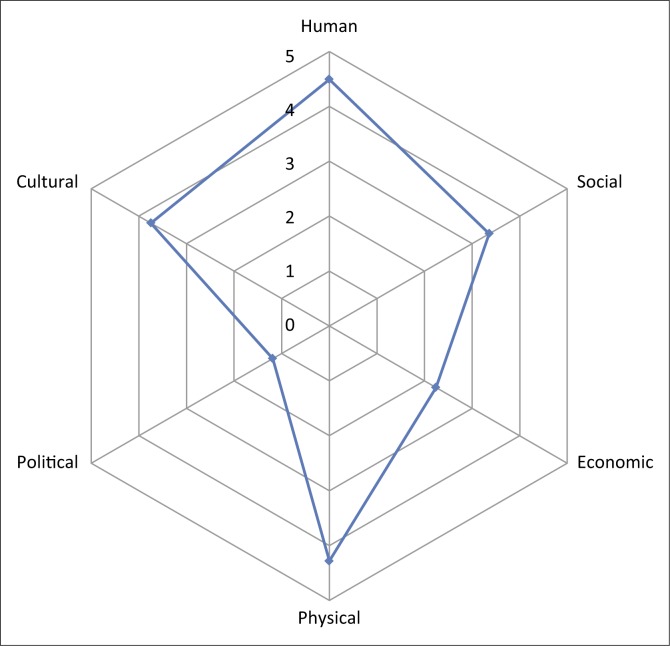
Overall ratings of capitals.

Finally, the multi-variant factors of cultural capital indicated as moderate (50), high (27) and very high (189) accounted to 266 respondents valued the respect shown to gender issues in South Africa. The respondents expressed that there was professionalism in the workspace shown by both the employer and the employee. They as women were respected and their rights were observed. This was in contrast to many situations in their home countries. One woman from Ghana was happy in South Africa, having migrated 17 years ago because she was escaping genital mutilation. Work ethics and traditional beliefs were also rated to be good as cultural issues were clearly defined in the country’s constitution in South Africa. Hence, the ratings were as follows: (1) respect for authority, (2) gender issues, (3) work ethics and (4) traditional beliefs. Figure 3 summarises the livelihood capitals that the respondents employed in South Africa to cope and adapt.

Human (education, health, knowledge and skills, and capacity to work), social (networks and connections; relations of trust and mutual support; informal and formal networks), economic (salary/wages/income, savings, credit, pension, marriage, and *stokvels* and burial societies), physical (shelter/houses, amenities [basic services], communication services (vehicles, transport networks), technology (Internet, emails, cellphones, etc.), political (relationships with political authorities in South Africa, political networks from home country, interaction with political structures locally and business relations with politicians) and cultural (gender issues, traditional beliefs, work ethics and respect for authority) livelihood capitals were very significant for the survival of women migrants. These livelihood capitals have an implication directly and indirectly; when we look at direct implication, the migrant women need to have education and health service to survive in day-to-day activities of their life. Basically, they need also to sustain economically at least to cover house rent, food, communicate with family and assist the family. Furthermore, they need to adapt, respect and live with the culture of the host nation. The indirect implication of lives on, the livelihood of migrant women affects, if they are not economically strong, they are not motivated to return to their home empty-handed. Some of them stated also that they are willing to return to their home but the political turmoil of their home country (e.g. Somalia and DRC) makes it difficult for them to return. From the cultural point of view, some of the women stated that they do not have independence (like from Somalia [one], Ethiopia [three] and Eritrea [one]), economic freedom and decision on resources and others in their home country; once they are used to stay in South Africa, it would be difficult for them to return to their home; it will not be easy to adjust their home country situation, especially when related to gender stereotype.

The survey interviews were complemented also by open questions where the informants were given a chance to elaborate further on some of the capitals as a coping mechanism. This indicates that there is not a magic bullet means of livelihood capital to adapt and cope; migrant women utilised different means of surviving mechanisms. See Box 1 for some of the responses.

Box 1 Network support mechanisms.

**Family support**

My family is motivating me to work harder. My family from home sends me some money. They keep me happy and motivate me to keep going. My sister is always there providing me with food. My brother assisted when I came to South Africa, sent me to school and assisted me to start the salon business. My uncle assisted me with accommodation and basic things like food.

**Religious support**

The church is providing spiritual and emotional support. At times people from church give me food. They assist when I have problems. Some church members lend me money, and they help with refereeing me to places where I can get employment. I have managed to link up with a group from church to be able to buy goods in bulk so that we can sell at competitive prices. At times when one moves to another town, church members connect you to the church members in a new town. There are always people ready to welcome you and make you feel at home.

**Humanitarian support**

During bereavements, many foreigners, especially my compatriots, come together for support purposes and financial support. They organise financial resources and transport to repatriate the body back home as many of us foreigners like burying our fellow countrymen back home. When my husband was shot in an armed robbery, I had my fellow from my country supporting me with all the burial arrangement and they helped me sort out my husband’s estate. That is why I am surviving from his estate as he had a lot of money acquired through his business.

The neighbours give me food. They look after my property when I decide to go visit my home. They help me with the translation of their various local languages. They also teach me the languages so that I integrate fast into society.

## Conclusion

South Africa is a country that hosts many South–South international migrants. Knowing about the socio-economic mechanisms and other factors contributing to the coping and adaptation mechanisms in host countries brought some new insights to the expansion of social strategies that raised the resilience levels of migrant women. The findings showed that migrant women were resilient, independent and could resist and withstand the challenges of migration and cope and adapt. The study found that human capital ranked the highest, followed by physical, cultural, social, economic and political capitals. This implies that the livelihood capital has an implication; the migrant women need to have education and health services to survive in day-to-day activities of their life as human capital. They need also to sustain economically at least to cover house rent, food, communicate with family and assist the family as economic and physical capitals. Furthermore, they need to adapt, respect and live with the culture of the host nation with harmony and conducive environment as social, cultural and political capitals. For women migrants, it will not be easy to survive without assistance of the entire system.

## Recommendations

This study recommends that training, education and awareness campaigns need to be implemented at grassroots levels so that local inhabitants can understand international migration and the benefits it brings to host countries in order to reduce xenophobic sentiments. The migrant women fostered a favourable economic atmosphere and therefore it is in the host government’s hands to become proactive and capitalise on innovative ideas brought into the country by migrants. This could be done through providing more opportunities through good and relevant education systems and by proactively learning from other countries that have managed to create valuable human capital base. For instance, the United States of America benefited using migrants for their economic and political gain, for example, recently a Somali ex-refugee woman was elected to represent in Congress.

## Footnotes

1 A *Stokvel* is an informal savings pool or syndicate, usually among black people, in which funds are contributed in rotation, allowing participants lump sums for family needs, especially funerals (Oxford Dictionaries 2017)
